# Management of an Unusual Tracheoesophageal Party Wall Foreign Body

**Published:** 2019-01

**Authors:** Kalaiarasi Raja, Sithananda-Kumar Venkatesan, Sivaraman Ganesan, Arun Alexander

**Affiliations:** 1 *Department of ENT, Sri Lakshmi Narayana Institute of Medical Sciences, Puducherry, India.*; 2 *Department of ENT, Pondicherry Institute of Medical Sciences, Puducherry, India.*; 3 *Department of Otorhinolaryngology, JIPMER, Puducherry 605009, India.*

**Keywords:** Esophagus, Foreign body, Neck, Trachea

## Abstract

**Introduction:**

Penetration injury to the neck constitutes 5–10% of all trauma cases. Penetration of a foreign body into the trachea with subsequent impaction into the tracheoesophageal party wall is extremely rare. We present a patient with an unusual penetrating injury of the neck caused by a metallic foreign body embedded into the tracheoesophageal party wall, and its management.

**Case Report:**

A 35-year-old male presented to the emergency department with a history of accidental penetrating injury on his neck, with severe pain and bleeding from the wound entry site. On neck examination, there was an open wound, 0.5 × 0.5 cm in size, in the lower-third anterior aspect of the neck with surrounding neck swelling and tenderness. Computed tomography showed a radio-dense foreign body lodged in the tracheoesophageal party wall at the level of the second and third tracheal rings, which was removed successfully.

**Conclusion:**

Impacted foreign body following a penetrating wound in the neck needs considerable assessment and appropriate management.

## Introduction

Impacted foreign body in the neck following a penetrating injury is often unpredictable. Penetration of a foreign body into the trachea with subsequent impaction into the tracheoesophageal party wall is extremely rare, and there are no previous reports in the medical literature. Although the condition is rare, it can lead to several serious complications such as tracheal collapse, airway compromise, laryngotracheal separation, injury to the major neck vessels, or retropharyngeal abscess (1). The clinical presentations may vary from severe stridor or bleeding from the major neck vessels to persistent pain and foreign body sensation. The most challenging part is the surgical removal of such foreign bodies. We present a case of unusual penetrating neck injury caused by a metallic foreign body embedded into the tracheoesophageal party wall, and its management.

## Case Report

**Fig 1:**
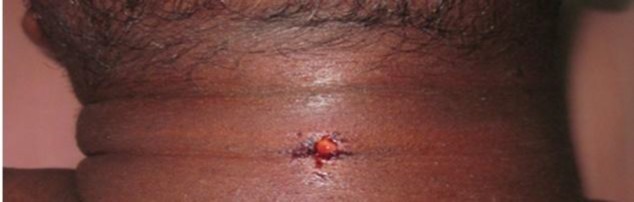
Picture showing penetrating neck injury entry site

A 35-year-old male carpenter presented to our emergency department with a history of accidental penetrating injury on his neck caused by a piece of metal breaking from a saw when he was using and hit him on his neck. The patient had severe pain and bleeding from the wound entry site, which was aggravated on swallowing, speaking and on moving the neck. There was no history of difficulty in breathing, change in voice or cough.On clinical examination, the patient was conscious, well oriented and hemodynamically stable without any neurological deficit. On neck examination, there was an open wound, about 0.5 × 0.5 cm in size, in the lower third anterior aspect of the neck with surrounding neck swelling and tenderness (Fig.1). Subcutaneous emphysema was present, extending from the inferior border of the mandible to the clavicle. Videolaryngoscopic examination showed normal vocal cord mobility.

**Fig 2:**
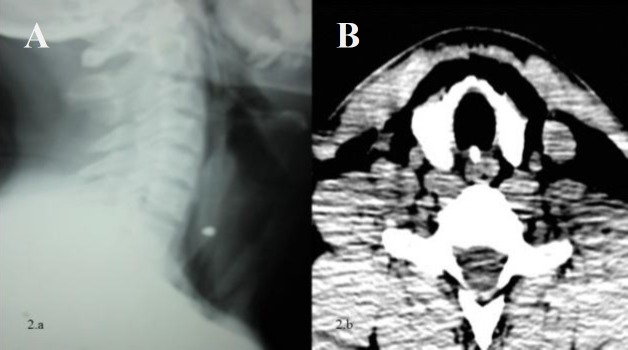
a) X-ray soft tissue neck lateral view showing the radio-opaque foreign body located in the tracheoesophageal party wall at the C6–C7 level with air lucency noted in the prevertebral space. b) CECT scan neck showing radio-dense metallic foreign body located in the trachea-esophageal party wall with air dissection in the subcutaneous plane

X-rays of the cervical region and chest were taken immediately in the anteroposterior and lateral view. A soft tissue neck (STN) lateral view revealed a radiopaque foreign body shadow at the level of the C6–C7 vertebral body level (Fig.2a). This was further confirmed with contrast enhanced computed tomography (CECT) of the neck with fine 1-mm cuts, which also showed that the radio-dense foreign body lodged in the tracheoesophageal party wall at the level of second and third tracheal rings, corresponding to the C7 vertebral body (Fig.2b).

**Fig 3:**
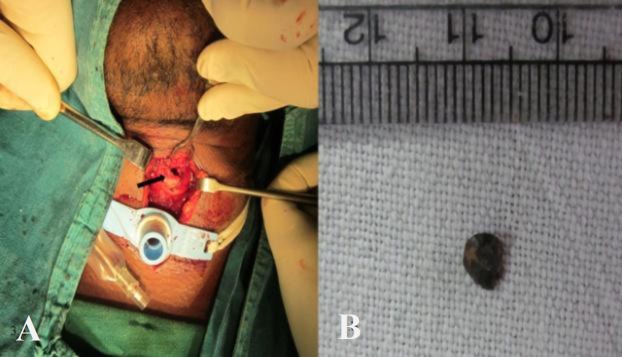
a) Image showing neck exploration by extending tracheostomy skin incision superiorly and tracheal entry site of the foreign body (arrow).  b) Removed metallic foreign body

The patient was taken for immediate surgical exploration. A low tracheostomy was performed under local anesthesia at the level of the fourth tracheal ring, which was below the level of the penetrating wound. A Portex cuffed tracheostomy tube (size 7.5) was inserted, and general anesthesia (GA) was administered through the tracheostomy tube. The vertical incision for tracheostomy was extended superiorly up to the cricoid cartilage. The strap muscles were retracted, and the thyroid isthmus was retracted above. The small entry point was found on the anterior wall of the second tracheal ring (Fig.3a). We opened the second tracheal ring at the site of entry in a vertical fashion. The second tracheal ring was opened in an open-book fashion, and the foreign body was visualized embedded in the party wall between the esophagus and the trachea. The foreign body was gently removed, avoiding accidental slippage into the distal airway (Fig.3b). 

Hemostasis was achieved and the opened second tracheal ring was approximated atraumatically using Prolene 3-0. The wound was closed in two layers. A nasogastric tube was inserted and removed on postoperative Day 2 (POD). On POD5, the patient was decannulated successfully and discharged from hospital without any complications.

## Discussion

Penetrating trauma to the neck can be caused by many kinds of materials, such as a knife, shotgun, pencil or glass fragments (2,3). In our case, it was a metallic fragment embedded in the party wall following a penetrating neck injury. Penetrating neck injury is a serious condition with high morbidity and potential mortality, constituting 5–10% of all trauma cases (4). In the neck region, there are vital structures which are at risk, and hence an early diagnosis is essential to avoid complications. The priority is establishing airway and hemodynamic stability in the patient, then a detailed assessment of the severity and site of the penetrating neck injury is required. The penetrated foreign body can lodge into the major vessels, trachea, esophagus and neurovascular bundle. In our case, it had become lodged in the party wall, a site which is difficult to access presenting a therapeutic dilemma. Although the entry wound was small, making the injury look insignificant, the foreign body lodged into the vital structure of the neck, and hence immediate neck exploration was planned.

Preoperative imaging studies such as radiographs, computed tomography scan, and magnetic resonance imaging help to locate the site of injury and its relation to the surrounding structures which help in surgical management. In our case, an STN lateral view radiograph revealed the site of the foreign body. Because the patient was hemodynamically stable, CECT neck with fine 1-mm cuts, which is the investigation of choice, was performed to identify the exact location of the foreign body and the direction of penetration to guide our surgical approach successfully.

The decision for early neck exploration was made based on imaging studies. We initially performed a low tracheostomy under local anaesthesia, through which GA was given. We avoided intubation, as it might cause dislodgement of the foreign body into the lower airway and would have obscured the impacted foreign body site. We followed the entry wound site, and the impacted foreign body was removed successfully without causing any damage to the vital structures. Immediate neck exploration is mandatory to avoid complications such as migration of the foreign body, granuloma and abscess formation (5). The trajectory of the foreign body penetration must be kept in mind to predict the site of impaction. A high-velocity injury can cause severe damage to the vital structures. The platysma serves as a landmark, as violation of this layer can lead to injury to deeper more vital structures (6).

## Conclusion

Penetrating injury to the neck is an uncommon but potentially life-threatening condition. Impacted foreign body following a penetrating wound in the neck needs a great deal of assessment and appropriate management. Although the entry wound might look insignificant, the underlying damage could be much graver, and hence immediate neck exploration is mandatory.

## References

[1] Poluri A, Singh B, Sperling N, Har-El G, Lucente FE (2000). Retropharyngeal abscess secondary to penetrating foreign bodies. J Craniomaxillofac Surg.

[2] Rothschild MA, Karger B, Schneider V (2001). Puncture wounds caused by glass mistaken for with stab wounds with a knife. Forensic Sci Int.

[3] Pickles JM (1988). Retropharyngeal abscess complicating a neck wound (a case report). J Laryngol Otol.

[4] Gulia J, Yadav S, Singh K, Khaowas A (2009). Penetrating Neck Injury: Report Of Two Cases. Internet J Emerg Med.

[5] Mathai J, Ahmed S, Pushpakumari KP, Reynolds AM (2007). Removal of a metallic foreign body in the neck with a magnet: A case report. Indian J Otolaryngol Head Neck Surg.

[6] Imokawa H, Tazawa T, Sugiura N, Oyake D, Yosino K (2003). Penetrating neck injuries involving wooden foreign bodies: the role of MRI and the misinterpretation of CT images. Auris Nasus Larynx.

